# Cooperative Switching in Nanofibers of Azobenzene Oligomers

**DOI:** 10.1038/srep25605

**Published:** 2016-05-10

**Authors:** Christopher Weber, Tobias Liebig, Manuel Gensler, Anton Zykov, Linus Pithan, Jürgen P. Rabe, Stefan Hecht, David Bléger, Stefan Kowarik

**Affiliations:** 1 Department of Physics, Humboldt-Universität zu Berlin, 12489 Berlin, Germany; 2 IRIS Adlershof, Humboldt-Universität zu Berlin, 12489 Berlin, Germany; 3 Department of Chemistry, Humboldt-Universität zu Berlin, 12489 Berlin, Germany

## Abstract

Next-generation molecular devices and machines demand the integration of molecular switches into hierarchical assemblies to amplify the response of the system from the molecular level to the meso- or macro-scale. Here, we demonstrate that multi-azobenzene oligomers can assemble to form robust supramolecular nanofibers in which they can be switched repeatedly between the *E*- and *Z*-configuration. While in isolated oligomers the azobenzene units undergo reversible photoisomerization independently, in the nanofibers they are coupled via intermolecular interactions and switch *cooperatively* as evidenced by unusual thermal and kinetic behavior. We find that the photoisomerization rate from the *Z*-isomer to the *E*-isomer depends on the fraction of *Z*-azobenzene in the nanofibers, and is increased by more than a factor of 4 in *Z*-rich fibers when compared to *E*-rich fibers. This demonstrates the great potential of coupling individual photochromic units for increasing their quantum efficiency in the solid state with potential relevance for actuation and sensing.

Synthetic photoresponsive molecules can be used as building blocks for a large variety of emerging applications ranging from molecular sensors^1^, energy^2^ and information^3^ storage devices to nano-optomechanical devices such as molecular motors^4^,^5^,^6^,^7^,^8^,^9^,^10^, artificial muscles^11^,^12^ and photoactuators^13^,^14^. For enabling these applications it is important to convert molecular nanoscale events into measurable effects at meso- and macro-scales by integrating molecular switches into well-defined hierarchical assemblies^15^,^16^,^17^. This can be achieved by both covalent bonding of monomeric switching units to form photoresponsive oligomers or polymers as well as their supramolecular assembly.

Within these densely packed supramolecular systems, it is important to control and, ideally, leverage the intra- and intermolecular interaction between switching units, because steric interaction, as well as excitonic and electronic coupling between adjacent molecular switches, alter the switching process^18^. Too strong coupling can completely suppress the photoresponse, both via simple steric hindrance or via excitonic coupling leading to delocalization of the excitation before the energy can be transferred to the nuclear coordinates^19^,^20^. However, coupling within a supramolecular assembly can also lead to positive cooperativity, that is, the chromophores do not switch independently from each other but exhibit emergent behavior, *e.g.*, by supporting switching cascades^21^,^22^,^23^. In biological systems, such cooperativity is commonly used to accelerate specific processes and to increase or amplify the outcome of reactions^24^.

In synthetic photoresponsive systems, it is an interesting question, if cooperative interaction between molecular switches could be used to amplify the impact of individual photoisomerization events. Ultimately, an array of cooperatively coupled molecular switches could act as a molecular amplifier that magnifies the power of an input signal at the nanoscale, such as absorption of a single photon, or a molecular recognition event into an output signal at the macroscopic scale, *e.g.*, by releasing stored internal energy corresponding to the energetic difference between two switching states. Possible applications include sensing devices, where cooperativity could be used to amplify single-molecule events at the nanoscale to readily measurable effects involving many molecules and thus to increase the sensitivity^25^,^26^.

However, there are only very few reports of artificial systems exhibiting cooperative switching, yet^27^,^28^,^29^. One problem is to design molecular systems in which the intermolecular coupling is not so strong as to impede reversible switching but strong enough to make the isomerization dependent on neighboring molecules. Azobenzene derivatives in particular are promising building blocks for cooperatively switching multicomponent molecular systems. This is because the isomerization from the stretched out *E*-isomer to the compact *Z*-isomer is accompanied by significant changes of geometry and dipole moment, which enables neighboring chromophores to interact during isomerization through steric or electronic coupling. Azobenzene polymers incorporating multiple covalently bonded azobenzenes in their main chain exhibit contractile motion upon UV-irradiation^30^,^31^. Recently, we developed a rigid rod polymer (**P1**) that incorporates more than 30 azobenzene units in its poly(*p*-phenylene)s (PPP) backbone^32^,^33^,^34^. The introduction of large dihedral angles in the **P1** backbone makes sure that the azobenzenes along the polymeric chain are electronically decoupled and photoisomerize with high efficiency^35^.

In this work, we show that azobenzene oligomers of type **P1** self-assemble into well-defined nanofibers and quantitatively analyze the switching kinetics of these linear, supramolecular assemblies. We find that the *Z* → *E* photoisomerization of azobenzenes in **P1** nanofibers is significantly slower at elevated temperatures, in contrast to isolated azobenzene oligomers where both thermal and optical *Z* → *E* isomerizations are faster at elevated temperatures. Further, we find that in the nanofibers the effective *Z* → *E* photoisomerization rate is increased by up to a factor of 4, depending on the fraction of azobenzenes in the *Z*-configuration. We attribute this to an increased photoisomerization efficiency due to cooperativity among individual switching units^36^.

## Results and Discussion

**Figure 1:**
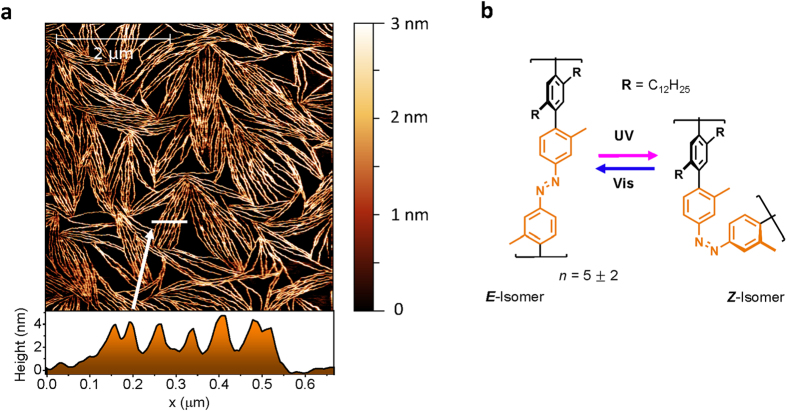
Sample morphology and molecular structure of P1. (**a**) AFM image showing a 5 × 5 μm^2^ region of a spin coated sample of **P1** oligomers with a height profile. (**b**) Chemical structure of **P1**.

Figure 1a displays an AFM image of a sample of azobenzene oligomers after deposition onto an oxidized silicon wafer via spin coating from a toluene solution. The SiO_2_ surface is covered by a fibrillar network. Single nanofibers exhibit a characteristic length in the range of 1–2 μm, a height of about 4 nm, and an apparent width of about 20 nm, which, due to the finite radius of the AFM tip (<10 nm)^37^, corresponds to a real width on the same order as the measured height. The typical spacing between the nanofibers is several 10 nm. Since the length of an isolated oligomer in the thermally stable *E*-configuration is only around 8 nm, the nanofibers are attributed to supramolecular aggregates consisting of many oligomers. Interestingly, only very few of the nanofibers on the SiO_2_ surface have a loose end, which indicates the formation of higher order aggregates such as in multiple helices^38^,^39^.

For the side-chain alignment we recently demonstrated that, in thin films, **P1** polymers can form coherently ordered nanodomains of interdigitating alkyl side-chains. X-ray diffraction measurements performed on a **P1** oligomer nanofiber sample, albeit at a higher nominal thickness, show one of the three Bragg reflections of **P1** polymers (see the Supplementary Information, Figure S2), indicating some coherent ordering in the nanofibers.

**Figure 2:**
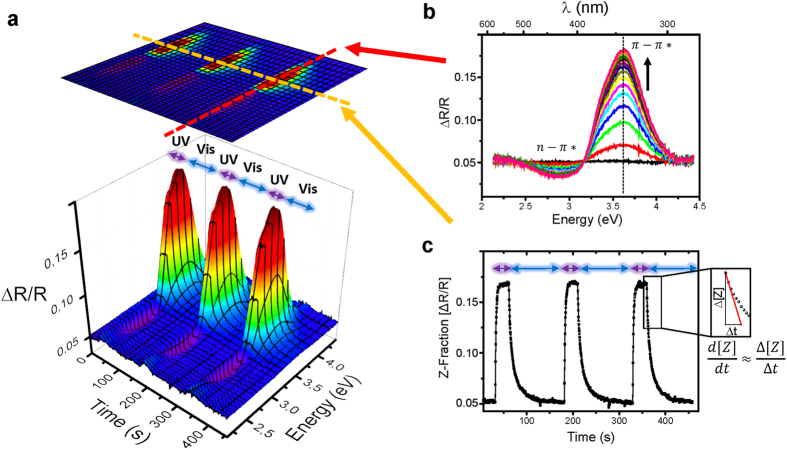
Differential reflectance spectroscopy (DRS) of P1-nanofibers during photoisomerization. (**a**) Reversible switching of an azobenzene nanofiber sample is apparent from a 3D graph of the optical differential reflectance during three switching cycles. Cross sections at fixed time as a function of the energy yield DRS spectra (**b**) and cross sections at 3.5 eV as a function of time allow for determination of the rate of *Z* → *E* isomerization (**c**).

The nanofibers persist even after many switching cycles as we checked with AFM (see the Supplementary Information, Figures S2 and S3). Furthermore, samples measured almost two years after preparation of the film show the same fibrillar structures. These findings underline the robustness of the nanofibers when stored under ambient conditions. Differential reflectance spectroscopy (DRS)^40^ provides an easy and efficient way to monitor in real-time the conversion of *E*-azobenzene to *Z*-azobenzene and *vice versa* in **P1** thin films. DRS measurements demonstrate that the azobenzene chromophores in the thin film can be switched reversibly back and forth with UV light and visible light, respectively. Figure 2a shows a 3D plot of the differential reflectance spectra Δ***R***/***R***, that is the change in reflectance divided by the initial reflectance, over time. When the UV LED is switched on for the first time at ***t*** = 30 s, the reflectance is increased in the energy range between 3.2 eV and 4.2 eV and is decreased in the energy range between 2.5 eV and 3.2 eV (see Fig. 2b). The increase of the reflectivity, that is positive Δ***R***/***R***, around 3.6 eV during *E* → *Z* photoisomerization can be rationalized with decreasing absorption in the π − π* absorption band of the *E* isomer. Conversely, in the region around 2.7 eV, the reflectivity is decreased as the n − π* band of the *Z*-isomer starts to absorb upon *E* → *Z* photoisomerization. Besides these large changes due to the changing fraction of *E* and *Z* populations with their respective absorption bands, only a small gradual blue-shift of the individual *E*-state π − π* and *Z*-state n − π* bands is observed with DRS, amounting to a total shift of about 10 meV (see SI).After the UV LED is switched off at ***t*** = 60 s, the reflectance returns to its initial value before the first UV-irradiation due to the white probe light. Figure 2c shows a cross section through Fig. 2a along the temporal axis at an energy of 3.5 eV.

The photoisomerization kinetics of the azobenzene chromophores can be extracted from the temporal evolution of the differential reflectance. To find the relation between the DRS signal and the photoisomerization kinetics, we simulated the DRS signal of a **P1** film on silicon covered with native oxide using the transfer matrix method (see the Supplementary Information, Figure S4)^41^. Our simulations show that the DRS signal Δ***R***/***R*** close to the π − π* absorption at 3.5 eV depends linearly on the fraction of *Z*-azobenzene, that is, the ensemble switching kinetics can be directly inferred from the DRS measurement. A significant amount of roughly 70% of the azobenzenes isomerizes after UV-irradiation with a power density of 110 mW/cm^2^, according to simulations of the DRS signal using realistic values of the geometry, packing densities and the optical constants of azobenzene in **P1** nanofibers (see SI).

The conversion process from *Z*-azobenzene to *E*-azobenzene can be described by the following rate equation


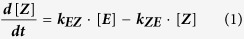


Here, [*E*] and [*Z*] denote the fraction of *E*- and *Z*-azobenzene, ***k***_***EZ***_ denotes the rate constant of the *E* → *Z* photoisomerization having contributions from both the UV-part of the Xe-lamp spectrum and the UV LED, and 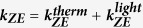 denotes the *Z* → *E* isomerization rate constant of azobenzenes having both thermal contributions and contributions from the Xe-lamp. The thermal contribution to the *Z* → *E* isomerization is, however, negligible in comparison with the optical contribution since pure thermal back-switching of azobenzenes typically takes several hours whereas the isomerization process we observe occurs within seconds.

The solution of eq. (1) is a monoexponential function with the apparent (effective) isomerization rate 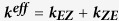. The effective isomerization rate *k*^*eff*^ can be determined experimentally from the real-time DRS data via


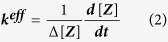


where 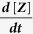 and [***Z***] are the velocity of the *Z* → *E* conversion and the fraction of *Z*-azobenzene at a certain time and Δ[***Z***] denotes the absolute difference between the high and low asymptotical level of *Z*-azobenzene fraction. In the following, we discuss the temperature and light-intensity dependence of the *Z* → *E* isomerization rate, that is, the initial decay of the time-dependent *Z*-azobenzene fraction.

**Figure 3:**
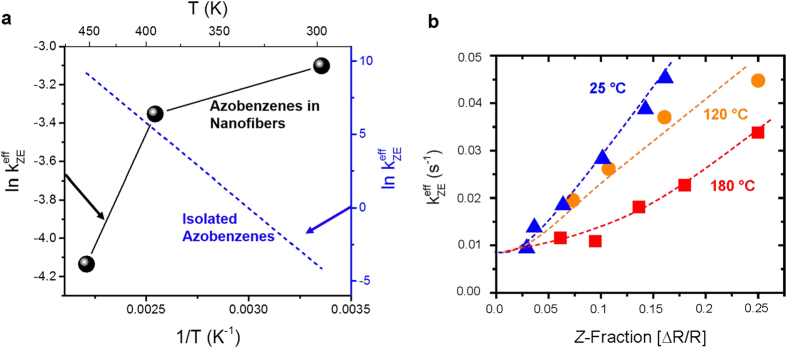
Apparent ensemble photoisomerization rates of P1-oligomers for different temperatures. (**a**) Arrhenius plot of the rate of the *Z* → *E* photoisomerization reaction measured at three different temperatures starting from the same level of *Z*-azobenzene fraction (≈0.15). The thermal behavior we find is in clear contradiction to the Arrhenius-type behavior of isolated azobenzenes (blue dotted line) that switch faster at higher temperatures. (**b**) Dependency of the *Z* → *E* photoisomerization rate *k*^*eff*^ on the fraction of *Z*-azobenzene in the film, plotted for three different temperatures (dotted lines to guide the eye), showing that the isomerization rate also depends on the fraction of *Z*-azobenzene.

Upon analyzing the kinetics of the *Z* → *E* photoisomerization, we find that the azobenzene chromophores in the nanofibers do not switch independently but exhibit cooperativity and a non-Arrhenius-type thermal switching behavior. The first indication is shown in Fig. 3a, where the temperature dependence of the measured effective isomerization rate ***k***^***eff***^ is plotted (see eq. 2). We find that the effective *Z* → *E* photoisomerization rate starting from the same fraction of *Z*-azobenzene decreases with increasing temperature. This behavior is diametrically opposed to the behavior of independently switching azobenzenes in solution, for which the thermal back-reaction across an energetic barrier on the order of 1 eV leads to an increasing ***k***^***eff***^ at higher temperatures^42^. The dotted line in Fig. 3a shows the typical Arrhenius behavior for a purely thermal process.

In addition to real-time measurements at different temperatures, we also measured the rate of the *Z* → *E* photoisomerization as a function of the starting fraction of *Z*-azobenzene in the nanofibers at three different temperatures (Fig. 3b). At room temperature, where the effect is the strongest, the effective photoisomerization rate can differ by more than a factor of 4 for different starting fractions of *Z-*azobenzene. This dependency of the ensemble switching kinetics of the azobenzene units on the isomeric state of the environment directly shows that the azobenzenes in the nanofibers do not switch independently but cooperatively. It is important to note that **P1**-type multi-azobenzene compounds, which are dissolved in cyclohexane at rather low concentration (≈10^−5^ mol/L) in order to avoid aggregation, do not switch cooperatively, that is, the individual azobenzene units within a single isolated macromolecule switch independently^35^.

**Figure 4:**
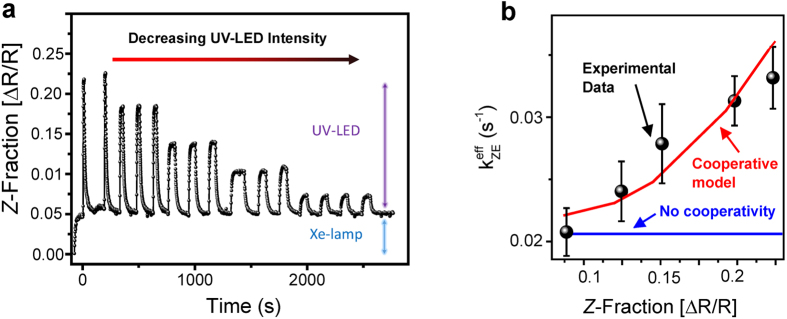
Photoisomerization kinetics for different UV-LED intensities. (**a**) Differential reflectance at 3.5 eV during several cycles of alternating irradiation with white light + UV light of varying intensities and white light. (**b**) Experimentally determined *Z* → *E* isomerization rates at different photostationary levels together with a simulation including cooperativity (red curve).

For a quantitative understanding of the cooperativity, we use an intentionally simple model to fit the isomerization kinetics. The real-time kinetics of back-switching from different fractions of *Z*-azobenzene and the extracted *Z*-dependent *k*^*eff*^ that have been used for the fitting are shown in Fig. 4a,b, respectively. Plotted in Fig. 4a is the fraction of *Z*-azobenzene corresponding to the differential reflectance during several cycles of UV-irradiation at room temperature with varying intensities. In addition, the sample is exposed to white probe light all the time. When the dark sample gets exposed to white probe light of the Xe-lamp, the *Z*-fraction increases and reaches a steady state. Additional UV-light from the LED (intensity I = 110, 40, 17, 7 and 1.7 mW/cm^2^ in the range 360 nm–370 nm) further increases the *Z*-fraction. With a higher LED UV-light intensity, a larger fraction of the azobenzenes is switched from *E* to *Z*. After the UV LED is switched off, the *Z*-fraction returns back to the level corresponding to exclusive irradiation with Xe-light.

For independent azobenzene chromophores, the effective rate of the *Z* → *E* back-isomerization 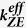 only depends on the spectrum of the Xe-lamp and the temperature and thus would be the same for all switching cycles shown in Fig. 4a. However, our real-time measurements show that the effective *Z* → *E* isomerization rate 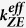 is significantly faster in switching cycles where a higher optical output power of the UV LED was used to induce *E* → *Z* isomerization. For example, the *Z* → *E* isomerization rate after UV irradiation with 110 mW/cm^2^ is about 50% faster than the *Z* → *E* isomerization rate after UV irradiation with 1.7 mW/cm^2^. The dependency of the back-switching rate on the *Z*-fraction cannot be described with the simple rate equation for isolated azobenzene moieties (1) because its solution is a monoexponential function with the time- and *Z*-independent rate 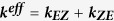. A simple modification to the rate equation (1) is to include a linear *Z*-dependency of the rates. In a first approximation, this introduces a dependency on the isomeric state of the microenvironment. We find that a modification of the *E* → Z isomerization rate alone cannot explain the observed switching behavior; however, both the kinetics and the photostationary levels of the real-time data can be fitted when modifying the back-switching rate as follows


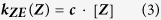


where ***c*** denotes a constant.

Inserting eq. (3) into eq. (1) the rate equation model includes cooperative behavior, that is, the *Z* → *E* isomerization is faster when there is a higher *Z-*azobenzene fraction in the film (see Fig. 4b). Note that we did not introduce additional model parameters, even though a refined model could include, *e.g.*, a constant offset to achieve a finite rate at [*Z*] = 0. According to the modified rate equation, also the *E* → *Z* isomerization is faster for higher fractions of *Z*-azobenzene. However, the deviation from the monoexponential process is significantly smaller for *E* → *Z* photoisomerization than in the case of the *Z* → *E* photoisomerization, since the contribution of the UV-LED to ***k***_***EZ***_ dominates eq. (1). In summary, we only need three parameters for the fits to our real-time data: ***k***_***EZ***_, ***c***, and a scaling parameter that scales [*Z*] to the DRS signal ([*Z*]/(Δ***R***/***R***) ≈ 4.1). The red line in Fig. 4b results from a fit of the solution of our modified rate equation to the experimental real-time data in *E* → *Z* switching direction for five different UV-intensities and for five corresponding *Z* → *E* back-switching processes. The good agreement of our simulation with the measured cooperative kinetics indicates that the linear *Z*-dependence of the *Z* → *E* isomerization rate, indeed, is a good approximation.

Based on the findings of a *Z*-dependent *Z* → *E* isomerization rate in nanofibers, we now discuss the origin of cooperativity, in particular, inter- and intramolecular interactions of the azobenzene chromophores. In principle, intramolecular interactions between azobenzene units can lead to cooperative switching behavior, and other isolated multi-azobenzene derivatives have been shown to exhibit cooperative switching in solution due to π stacking or electronic coupling between azobenzene moieties^28^. However, in the case of the rigid rod polymer **P1** the azobenzene chromophores within a single isolated molecule are electronically decoupled. In an earlier study no signs of cooperative switching have been found for isolated **P1**-type molecules in extremely diluted cyclohexane solution (≈10^−5^ mol/L), where no supramolecular aggregates are formed^35^. For comparison, our nanofiber samples were prepared from a solution with a much higher concentration of about 5 · 10^−2^ mol/L, whose high absorbance made spectroscopic measurements in solution difficult. In this earlier study, the isolated **P1**-type molecules exhibited Arrhenius-type thermal behavior and their switching kinetics followed a monoexponential function and thus did not depend on the fraction of *Z*-azobenzene. This clearly demonstrates that the cooperative behavior of **P1**-oligomers emerges due to their aggregation into nanofibers.

Intermolecular coupling of **P1** azobenzenes in nanofibers is possible, either via mechanical strain, changing free volume or dipole-dipole interactions. In the *Z*-configuration, azobenzene has a static dipole moment of about 3 Debye while azobenzene in the *E*-configuration has a dipole moment near zero^43^. Therefore static dipole-dipole coupling in a sample with a large fraction of *Z*-azobenzene could influence the back-switching kinetics of *Z*-azobenzene in the nanofibers. However, the interaction between molecular dipoles of *Z*-azobenzenes is probably not a dominating effect, because the enhancement starts even at the stages where the *Z*-azobenzene fraction is very low and the reacting *Z*-form is most likely to be surrounded by the *E*-form.

Another possible explanation for the observed enhancement of the effective *Z* → *E* photoisomerization rate at higher *Z*-azobenzene fraction could be an increased number of structural defects in the nanofibers when there are more azobenzenes in the bulky *Z*-configuration. A greater amount of free volume available to the switching units in the vicinity of defects could lead to a relative acceleration of the photoisomerization rate for a higher *Z*-azobenzene concentration and thus for a higher defect concentration. However, this theory cannot explain the finding of accelerated *Z* → *E* photoisomerization rates at lower temperatures. At higher temperatures beyond the melting temperature of the alkyl side-chain the number of gauge defects and other structural defects among the side-chain should be higher leading to an increased free volume per azobenzene unit. Thus, also the apparent photoisomerization rate should be larger at higher temperatures, which contradicts our experimental observations (see Fig. 3b).

Apart from electrostatic influences and structural defects, it is well known that mechanical strain can influence the isomerization kinetics of azobenzene^44^. The observed small shifts (Δ ≈ 10 meV) of the *Z*-form *n* − π* band around 2.7 eV and the *E*-from *π* − π* band around 3.6 eV can be attributed to strain, which varies with the amount of *Z*-isomer in the surrounding. The strain influence on the spectra is in agreement with the literature for other organic molecular systems^45^,^46^.

Tamaoki *et al*. demonstrated that for bridged bis-azobenzenes the *Z* → *E* isomerization is accelerated by orders of magnitude if the *Z*-isomer is strained^47^. Turanský *et al*. performed simulations of the photoinduced *Z* → *E* isomerization of stretched azobenzene and found that mechanical stretching can induce *Z* → *E* isomerization in the ground state and also favors the photoinduced *Z* → *E* isomerization via excited states^48^,^49^. These theoretical simulations performed for single azobenzenes can qualitatively explain our experimental findings for azobenzene oligomers in nanofibers. The *E* → *Z* isomerization induces steric strain in the nanofibers due to the non-planar geometry of the *Z*-isomer. Also, the *E* → *Z* isomerization of an azobenzene chromophore results in a more twisted, shorter oligomer, which in turn exerts pull along the backbone against forces from interlocked side-chain. Thus, an increase in *Z*-fraction leads to more strain and a greater mechanical stretching of the switched azobenzenes and therefore can explain the faster, *Z*-dependent photoinduced back-switching according to the simulations for single azobenzene molecules.

**Figure 5:**
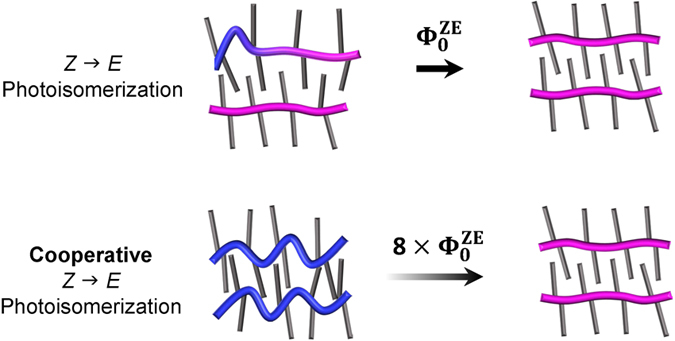
Illustration of the proposed switching mechanism. A higher *Z*-azobenzene concentration in the nanofibers corresponding to increased strain leads to an 8-fold increase of the quantum yield of photoisomerization 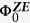.

Our second observation of slower back-switching at higher temperatures also can be explained by the temperature dependence of strain. At temperatures above 120–150 °C, roughly corresponding to the melting temperature of polyethylene, ordered alkyl side-chain domains involve more *gauche*-defects^50^, thereby decreasing the intermolecular interaction strength of azobenzene chromophores within different oligomers. This weaker coupling reduces the strain and thereby slows down isomerization rates to non-cooperative single molecule levels. An important implication of our findings is that intermolecular cooperativity can be used to increase the quantum yield of photoisomerization (see Fig. 5). According to our kinetic experiments and applying our modified rate equation model, the quantum yield for the *Z* → *E* photoisomerization of azobenzenes in the nanofibers can be increased by a factor of 8 (see Supplementary Information). A detailed comparison of the absolute values for quantum yield of **P1**-oligomers in nanofibers on a surface vs. the quantum yield of isolated **P1**-oligomers in solution is difficult because of the different experimental techniques and the comparatively large error bars of the optical constants of **P1**-oligomers in the nanofibers. Our estimate of a *Z* → *E* photoisomerization quantum yield Φ^*ZE*^ = 0.16 for high *Z*-content in the nanofiber indicates that even with positive cooperativity, switching in the nanofibers is slower than for azobenzene in solution, where a *Z* → *E* quantum yield (via n − π* excitation) of typically 0.4–0.6 is found^51^.

Theoretically, in multi-component molecular systems that utilize cooperativity it might be possible to raise the quantum yield of *Z* → *E* photoisomerization beyond unity, that is, to switch more than one azo-benzene unit with a single photon. While strain accelerates the *Z* → *E* photoisomerization, the strain thereby is also released and the cooperativity reduced in our system. Further tuning of the coupling between the chromophores, e.g., by introducing external mechanical strain or by using different coupling mechanisms would be needed to enable a switching cascade. This would be an important step towards a molecular amplifier, that is, a synthetic molecular device that converts input signals corresponding to single-molecule events at the nanoscale into a measurable signal involving many molecules at the macroscopic scale, *e.g.*, by leveraging internal energy, stored in the metastable *Z*-configuration of azobenzenes.

## Conclusions

In this work, we have shown that azobenzene oligomers form nanofibers with well-defined dimensions and shapes, having lengths of 1–2 μm, a height of 4 nm and a typical width on the same order. Whereas in diluted solution, isolated **P1**-type molecules switch independently, **P1** exhibits positive cooperativity when self-assembled into nanofibers. The *Z* → *E* isomerization rate depends approximately linearly on the fraction of *Z-*azobenzene within the film. In contrast to the common Arrhenius-type behavior of isolated molecular switches, the isomerization rate of azobenzenes in the nanofibers is faster at lower temperatures. We attribute this to the temperature dependency of the intermolecular coupling strength of the azobenzene chromophores via side-chain mediated strain. Moreover, we have shown that cooperativity of mechanically interlocked molecular switches can be utilized to increase the efficiency of the *Z* → *E* photoisomerization. Our findings are important for next-generation optomechanical devices and highly sensitive molecular sensors based on arrays of molecular switches.

## Methods

### Materials

The rigid-rod polymer **P1** incorporates azobenzene chromophores in a poly(*p-*phenylene) backbone and has two dodecyl side-chain per monomer (see Fig. 1). A crucial aspect of the design is the presence of *ortho-*methyl groups and therefore the introduction of large dihedral angles between the azobenzene units in order to decouple these units and break the electronic conjugation. This configuration makes it possible to attain a *Z*-rich photostationary state (PSS) upon irradiation with UV light. The polymers were synthesized as previously reported. The number average molecular weight M_n_ was 2900 g mol^-1^ (corresponding to five repeat units) and the sample had a polydispersity index of 1.45 as determined by GPC vs. polystyrene standards. The azobenzene oligomers can be switched from a thermodynamically stable linear and elongated conformation where the azobenzenes are in the *E-*configuration to a compact and kinked conformation with azobenzenes in the *Z*-configuration. Thin films of **P1** oligomers were cast from 25 mg/mL solution in toluene onto silicon wafers covered with native silicon oxide. The rotation speed of the bare substrate was set to 1500 rpm before the solution was dispensed.

### Measurements and Characterization

AFM measurements were carried out with a NanoWizard III microscope (JPK Instruments AG, Germany). The AFM was operated in air at room temperature in tapping mode with silicon cantilevers (AC240TS, Olympus Corporation, Japan, spring constant 2 N/m). For UV/Vis spectroscopy, we used a setup consisting of a 75 W Xenon Lamp (LOT-QuantumDesign, GmbH, Germany), a fiber with a reflectance probe (LOT-QuantumDesign GmbH, Germany), and a spectrometer consisting of a spectrograph (Acton Series, Princeton Instruments) and a cooled CCD (Andor). All UV/Vis spectroscopy measurements presented in this paper have been performed under atmospheric pressure. *E* → *Z* isomerization of **P1** was induced with a 365 nm high power UV LED (Thorlabs) with variable optical output and a FWHM of 12 nm. The light-intensity on the sample surface was measured with a thermal power sensor (Thorlabs).

## Additional Information

**How to cite this article**: Weber, C. *et al*. Cooperative Switching in Nanofibers of Azobenzene Oligomers. *Sci. Rep.* **6**, 25605; doi: 10.1038/srep25605 (2016).

## Supplementary Material

Supplementary Information

## References

[b1] Natali M. & Giordani S. Molecular switches as photocontrollable ‘smart’ receptors. Chem. Soc. Rev. 41, 4010–4029 (2012). 22426200 10.1039/c2cs35015g

[b2] Kucharski T. J., Tian Y., Akbulatov S. & Boulatov R. Chemical solutions for the closed-cycle storage of solar energy. Energy Environ. Sci. 4, 4449–4472 (2011).

[b3] Liu Z. F., Hashimoto K. & Fujishima A. Photoelectrochemical information storage using an azobenzene derivative. Nature 347, 658–660 (1990).

[b4] Yu Y., Nakano M. & Ikeda T. Photomechanics: directed bending of a polymer film by light. Nature 425, 145 (2003). 12968169 10.1038/425145a

[b5] Muraoka T., Kinbara K. & Aida T. Mechanical twisting of a guest by a photoresponsive host. Nature 440, 512–515 (2006). 16554815 10.1038/nature04635

[b6] Kay E. R., Leigh D. A. & Zerbetto F. Synthetic molecular motors and mechanical machines. Angew. Chem. Int. Ed. 46, 72–191 (2007). 10.1002/anie.200504313 17133632

[b7] Yamada M. . Photomobile polymer materials: towards light-driven plastic motors. Angew. Chem. Int. Ed. 47, 4986–4988 (2008). 10.1002/anie.200800760 18523940

[b8] Pathem B. K., Claridge S. A., Zheng Y. B. & Weiss P. S. Molecular Switches and Motors on Surfaces. Annu. Rev. Phys. Chem. 64, 605–630 (2013). 23331305 10.1146/annurev-physchem-040412-110045

[b9] Fahrenbach A. C. . Organic switches for surfaces and devices. Adv. Mater. 25, 331–348 (2013). 22933356 10.1002/adma.201201912

[b10] Uchida E., Azumi R. & Norikane Y. Light-induced crawling of crystals on a glass surface. Nat. Commun. 6, 7310, doi: 10.1038/ncomms8310 (2015). 26084483 PMC4557305

[b11] Browne W. R. & Feringa B. L. Making molecular machines work. Nat. Nanotechnol. 1, 25–35 (2006). 18654138 10.1038/nnano.2006.45

[b12] Coskun A., Banaszak M., Astumian R. D., Stoddart J. F. & Grzybowski B. A. Great expectations: can artificial molecular machines deliver on their promise? Chem. Soc. Rev. 41, 19–30 (2012). 22116531 10.1039/c1cs15262a

[b13] Juluri B. K. . A Mechanical Actuator Driven Electrochemically by Artificial Molecular Muscles. ACS Nano 3, 291–300 (2009). 19236063 10.1021/nn8002373

[b14] van Oosten C. L., Bastiaansen C. W. M. & Broer D. J. Printed artificial cilia from liquid-crystal network actuators modularly driven by light. Nat. Mater. 8, 677–682 (2009). 19561599 10.1038/nmat2487

[b15] Abendroth J. M., Bushuyev O. S., Weiss P. S. & Barrett C. J. Controlling Motion at the Nanoscale: Rise of the Molecular Machines. ACS Nano 9, 7746–7768 (2015). 26172380 10.1021/acsnano.5b03367

[b16] Klajn R. Immobilized azobenzenes for the construction of photoresponsive materials. Pure Appl. Chem. 82, 2247–2279 (2010).

[b17] Mathews M. . Light-driven reversible handedness inversion in self-organized helical superstructures. J. Am. Chem. Soc. 132, 18361–18366 (2010). 21126075 10.1021/ja108437n

[b18] Warren S. C., Guney-Altay O. & Grzybowski B. A. Responsive and Nonequilibrium Nanomaterials. J. Phys. Chem. Lett. 3, 2103–2111 (2012).

[b19] Gahl C. . Structure and excitonic coupling in self-assembled monolayers of azobenzene-functionalized alkanethiols. J. Am. Chem. Soc. 132, 1831–1838 (2010). 20099853 10.1021/ja903636q

[b20] Utecht M., Klamroth T. & Saalfrank P. Optical absorption and excitonic coupling in azobenzenes forming self-assembled monolayers: a study based on density functional theory. Phys. Chem. Chem. Phys. 13, 21608–21614 (2011). 22071571 10.1039/c1cp22793a

[b21] Zheng Y. B. . Photoresponsive molecules in well-defined nanoscale environments. Adv. Mater. 25, 302–312 (2013). 22933316 10.1002/adma.201201532

[b22] Pramanik S., De S. & Schmittel M. A trio of nanoswitches in redox-potential controlled communication. Chem. Commun. 50, 13254–13257 (2014). 10.1039/c4cc05773b 25227112

[b23] Pramanik S., De S. & Schmittel M. Bidirectional Chemical Communication between Nanomechanical Switches. Angew. Chemie Int. Ed. 53, 4709–4713 (2014). 10.1002/anie.201400804 24668761

[b24] Whitty A. Cooperativity and biological complexity. Nat. Chem. Biol. 4, 435–439 (2008). 18641616 10.1038/nchembio0808-435

[b25] Marsella M. J. & Swager T. M. Designing conducting polymer-based sensors: selective ionochromic response in crown ether-containing polythiophenes. J. Am. Chem. Soc. 115, 12214–12215 (1993).

[b26] Zhou Q. & Swager T. M. Method for enhancing the sensitivity of fluorescent chemosensors: energy migration in conjugated polymers. J. Am. Chem. Soc. 117, 7017–7018 (1995).

[b27] Pace G. . Cooperative light-induced molecular movements of highly ordered azobenzene self-assembled monolayers. Proc. Natl. Acad. Sci. USA 104, 9937–9942 (2007). 17535889 10.1073/pnas.0703748104 PMC1891213

[b28] Yu Z. & Hecht S. Cooperative Switching Events in Azobenzene Foldamer Denaturation. Chem. Eur. J. 18, 10519–10524 (2012). 22782860 10.1002/chem.201201624

[b29] Ray D., Foy J. T., Hughes R. P. & Aprahamian I. A switching cascade of hydrazone-based rotary switches through coordination-coupled proton relays. Nat. Chem. 4, 757–762 (2012). 22914198 10.1038/nchem.1408

[b30] Hugel T. . Single-molecule optomechanical cycle. Science. 296, 1103–1106 (2002). 12004125 10.1126/science.1069856

[b31] Holland N. B. . Single Molecule Force Spectroscopy of Azobenzene Polymers: Switching Elasticity of Single Photochromic Macromolecules. Macromolecules 36, 2015–2023 (2003).

[b32] Bléger D. . Light-orchestrated macromolecular ‘accordions’: reversible photoinduced shrinking of rigid-rod polymers. Angew. Chem. Int. Ed. 50, 12559–12563 (2011). 10.1002/anie.201106879 22114009

[b33] Weber C. . Light-Controlled ‘Molecular Zippers’ Based on Azobenzene Main Chain Polymers. Macromolecules 48, 1531–1537 (2015).

[b34] Lee C., Liebig T., Hecht S., Bléger D. & Rabe J. P. Light-Induced Contraction and Extension of Single Macromolecules on a Modified Graphite Surface. ACS Nano 8, 11987–11993 (2014). 25345562 10.1021/nn505325w

[b35] Bléger D. . Electronic decoupling approach to quantitative photoswitching in linear multiazobenzene architectures. J. Phys. Chem. B 115, 9930–9940 (2011). 21749103 10.1021/jp2044114

[b36] Sekkat Z. & Knoll W. Photoreactive Organic thin Films in the Light of Bound Electromagnetic Waves Ch. 2 (Academic Press, 2002).

[b37] Samorí P., Francke V., Mangel T., Müllen K. & Rabe J. P. Poly-para-phenylene-ethynylene assemblies for a potential molecular nanowire: an SFM study. Opt. Mater. (Amst). 9, 390–393 (1998).

[b38] Böttcher C. . Double-helical ultrastructure of polycationic dendronized polymers determined by single-particle cryo-TEM. Chem. - A Eur. J. 11, 2923–2928 (2005). 10.1002/chem.200401145 15736148

[b39] Weiss J., Jahnke E., Severin N., Rabe J. P. & Frauenrath H. Consecutive Conformational Transitions and Deaggregation of Multiple-Helical Poly(diacetylene)s. Nano Lett. 8, 1660–1666 (2008). 18462005 10.1021/nl080478h

[b40] Forker R., Gruenewald M. & Fritz T. Optical differential reflectance spectroscopy on thin molecular films. Annu. Rep. Prog. Chem., Sect. C Phys. Chem. 108, 34–68 (2012).

[b41] Burkhard G. F., Hoke E. T. & McGehee M. D. Accounting for interference, scattering, and electrode absorption to make accurate internal quantum efficiency measurements in organic and other thin solar cells. Adv. Mater. 22, 3293–3297 (2010). 20517871 10.1002/adma.201000883

[b42] Gegiou D., Muszkat K. A. & Fischer E. Temperature dependence of photoisomerization. V. Effect of substituents on the photoisomerization of stilbenes and azobenzenes. J. Am. Chem. Soc. 90, 3907–3918 (1968).

[b43] Bergmann E. & Weizmann A. Dipole Moments as a Tool in the Determination of Structure. Chem. Rev. 29, 553–592 (1941).

[b44] Yang Q.-Z. . A molecular force probe. Nat. Nanotechnol. 4, 302–306 (2009). 19421215 10.1038/nnano.2009.55

[b45] Helzel J., Jankowski S., El Helou M., Witte G. & Heimbrodt W. Temperature dependent optical properties of pentacene films on zinc oxide. Appl. Phys. Lett. 99, 211102, doi: 10.1063/1.3663863 (2011).

[b46] Li Y. *et al. In-situ* observation of nucleation processes during organic semiconductor thin film deposition and strain-stabilization of metastable states arXiv:1511.00085 (2016). 10.1038/srep32620 PMC5013491 27600905

[b47] Tamaoki N., Koseki K. & Yamaoka T. [2.2](4,4′)Azobenzenophane. Angew. Chem. Int. Ed. 29, 105–106 (1990).

[b48] Turanský R., Konôpka M., Doltsinis N. L., Štich I. & Marx D. Optical, mechanical, and opto-mechanical switching of anchored dithioazobenzene bridges. ChemPhysChem 11, 345–348 (2010). 19856368 10.1002/cphc.200900690

[b49] Turanský R., Konôpka M., Doltsinis N. L., Stich I. & Marx D. Switching of functionalized azobenzene suspended between gold tips by mechanochemical, photochemical, and opto-mechanical means. Phys. Chem. Chem. Phys. 12, 13922–13932 (2010). 20844786 10.1039/c0cp00588f

[b50] Serra S., Iarlori S., Tosatti E., Scandolo S. & Santoro G. Dynamical and thermal properties of polyethylene by ab initio simulation. Chem. Phys. Lett. 331, 339–345 (2000).

[b51] Dhammika Bandarab H. M. & Burdette S. C. Photoisomerization in different classes of azobenzene. Chem. Soc. Rev. 41, 1809–1825 (2012). 22008710 10.1039/c1cs15179g

