# Supplementary material for: Cooperative Switching in Nanofibers of Azobenzene Oligomers
Source: Sci Rep. 2016 May 10;6:25605. doi: 10.1038/srep25605 (PMC4861954; doi:10.1038/srep25605)
Supplement: Supplementary Information [file srep25605-s1.doc]

**Supplementary Information**

Cooperative Switching in Nanofibers of Azobenzene Oligomers

*Christopher Weber^1^, Tobias Liebig^1^, Manuel Gensler^1^, Anton Zykov^1^, Linus Pithan^1^, Jürgen P. Rabe^1,3^, Stefan Hecht^2,3^, David Bléger^2^, Stefan Kowarik^1^*

^1^ Department of Physics & ^2^ Department of Chemistry & ^3^ IRIS Adlershof, Humboldt-Universität zu Berlin, 12489 Berlin, Germany

**Table of Contents**

Gel permeation chromatography (GPC)……………..…..………...………………………....S2

X-ray diffraction and AFM measurement of samples with higher nominal thickness……….S2

AFM measurements before and after UV-irradiation ………………………………………..S5

Spectral shift during photoisomerization……………………………………………………..S5

Simulated differential reflectance spectroscopy (DRS)……………………………………....S6

Analytical fits of the real-time DRS data………...…….……………………………..............S9

DRS at elevated temperatures……………….……………………………... …….…...........S12

**Gel permeation chromatography (GPC)**

The synthesis of **P1** has been described elsewhere.^1^ However, in this work, we used a blend of shorter oligomers instead of polymers with > 30 repeating units. Figure S1 shows a gel permeation chromatography (GPC) measurement of a 1 mg/mL solution of azobenzene oligomers in tetrahydrofuran (THF). On the x-axis, the molar mass in g/mol is plotted on a logarithmic scale, whereas on the y-axis the intensity of the detected signal (UV absorbance, in red, refractive index, in green) is plotted. Five distinct peaks are visible in the range between 1 and 5 kg/mol, which can be attributed to short oligomers with lengths between 2 and 6 repeat units.

**
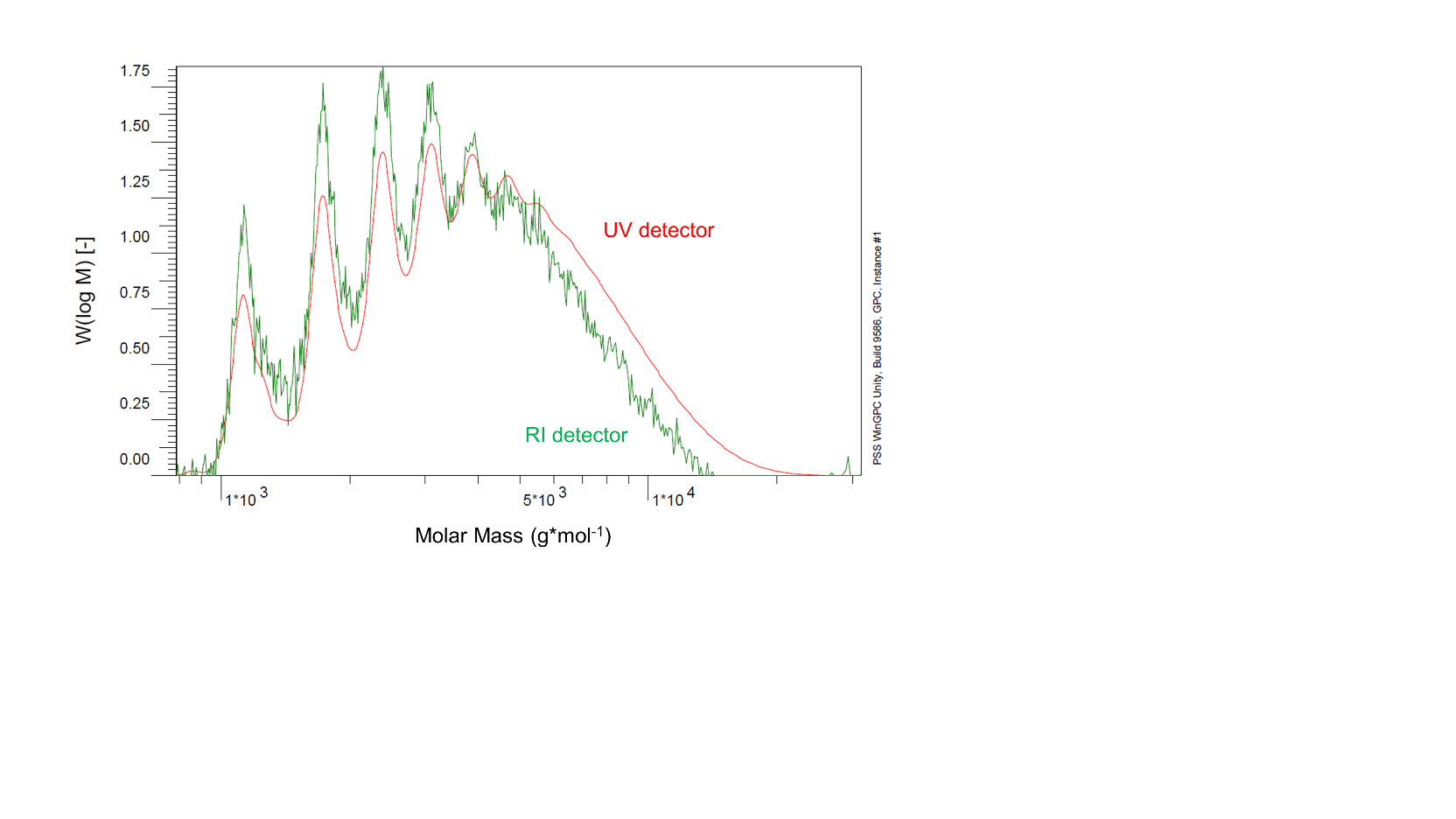
**

**Figure S1:** GPC measurement of a 1 g/L solution of azobenzene oligomers in THF.

**X-ray diffraction and AFM measurements of samples with higher nominal thickness**

To increase the scattering volume for X-ray diffraction measurements, we prepared samples with a higher nominal thickness. These samples were prepared by dispensing a drop of 25 mg/mL solution onto a resting substrate that was then rotated at 1500 rpm. Figure S2a displays an AFM image of a sample with higher nominal thickness (
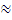
 16 nm).


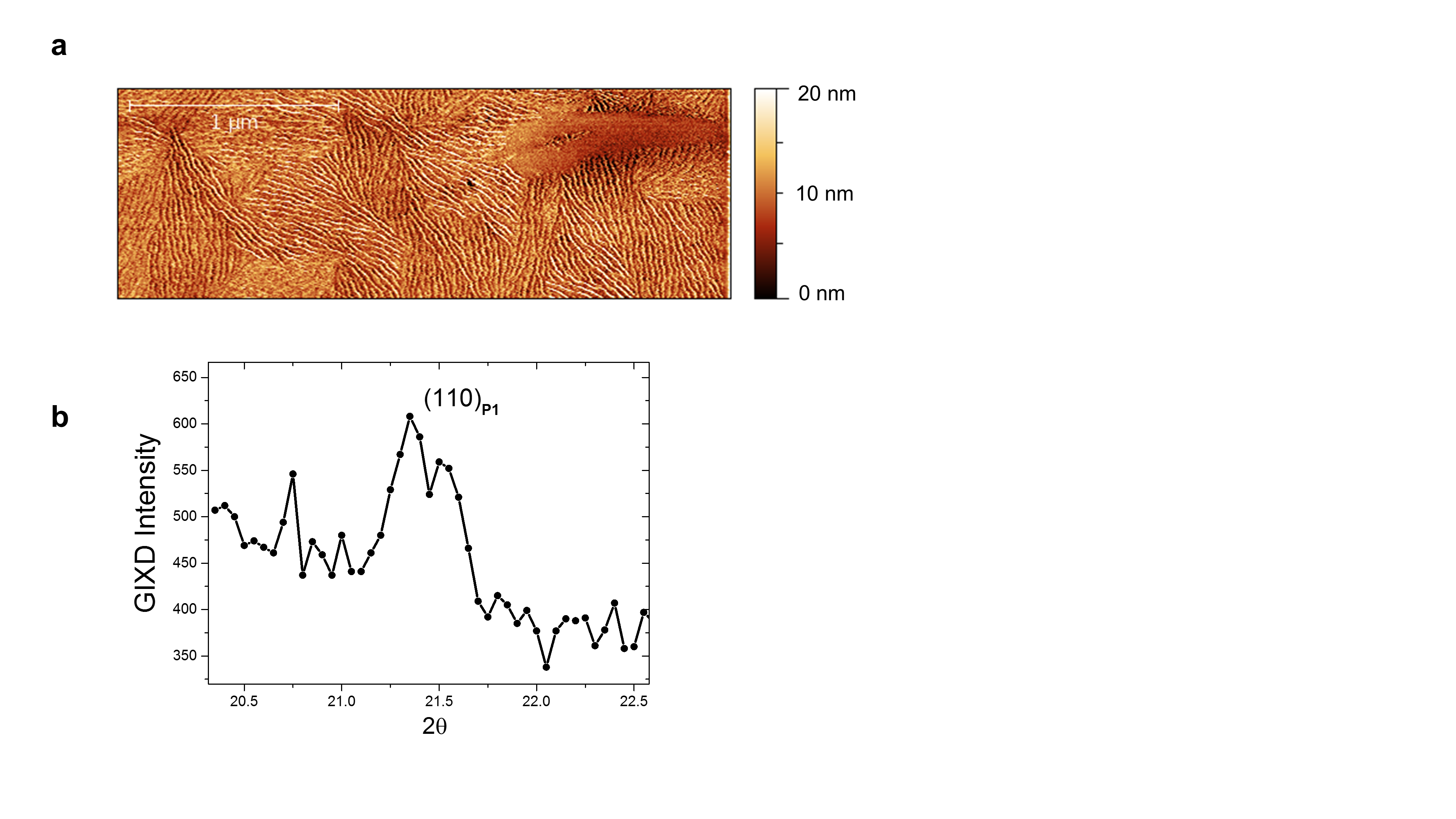


**Figure S2:** (a) AFM image recorded in height mode of a sample with higher nominal thickness that was used for X-ray diffraction measurements. The AFM image was acquired after numerous switching cycles on the previous day. (b) GIXD measurement of the same sample.

In comparison with AFM images of samples with lower coverages such as Figure 1a in the manuscript, the nanofibers are packed more densely on the oxidized silicon surface. We find fibrillar structures with lengths of up to 1 μm, a typical height of 10 nm and an apparent width of about 20 nm.

In an earlier study we found that in **P1** polymer samples the dodecyl side chains form crystalline nanodomains via interdigitation.^2^ GIXD measurements of **P1** oligomer samples have been performed with a *Rigaku* rotating anode using CuK_α_ X-ray radiation (λ=1.54 Å, α_i_ = 0.18°). Figure S2b shows the scattered x-ray intensity of a **P1** oligomer sample plotted against the in-plane detector angle 2θ. The peak between 21° and 22° can be attributed to the (110) in-plane Bragg reflection of the dodecyl side chains, following the notation of n-alkane crystals. This indicates the possibility that dodecyl side-chains also form crystalline nanodomains in nanofibers of **P1** oligomers in which the dodecyl chains point along the surface normal. It is possible to calculate a lower bound for the grain size
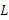
 of a molecular crystal using the Scherrer equation:


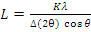
 (S1)

where
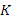
 is a dimensionless shape factor close to unity,
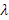
 is the wavelength of the x-rays,
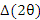
 is the FWHM of the Bragg reflection in radians after correcting the instrumental broadening and
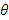
 is the Bragg angle. The measured FWHM of the Bragg reflection can be corrected from the instrumental broadening:


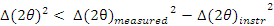
 (S2)

The instrumental resolution was about 0.32° and the FWHM of the (110) Bragg reflection was 0.45°. Inserting these values into eq. (S1) and (S2) gives a lower limit of the grain size in the direction of the (110) planes of 30 nm.

**AFM measurements before and after UV-irradiation**

We checked for light-induced structural transitions in the azobenzene oligomer thin films. Figure S3 shows AFM images taken before and after 5 min of UV-irradiation (30 mW/cm^2^).


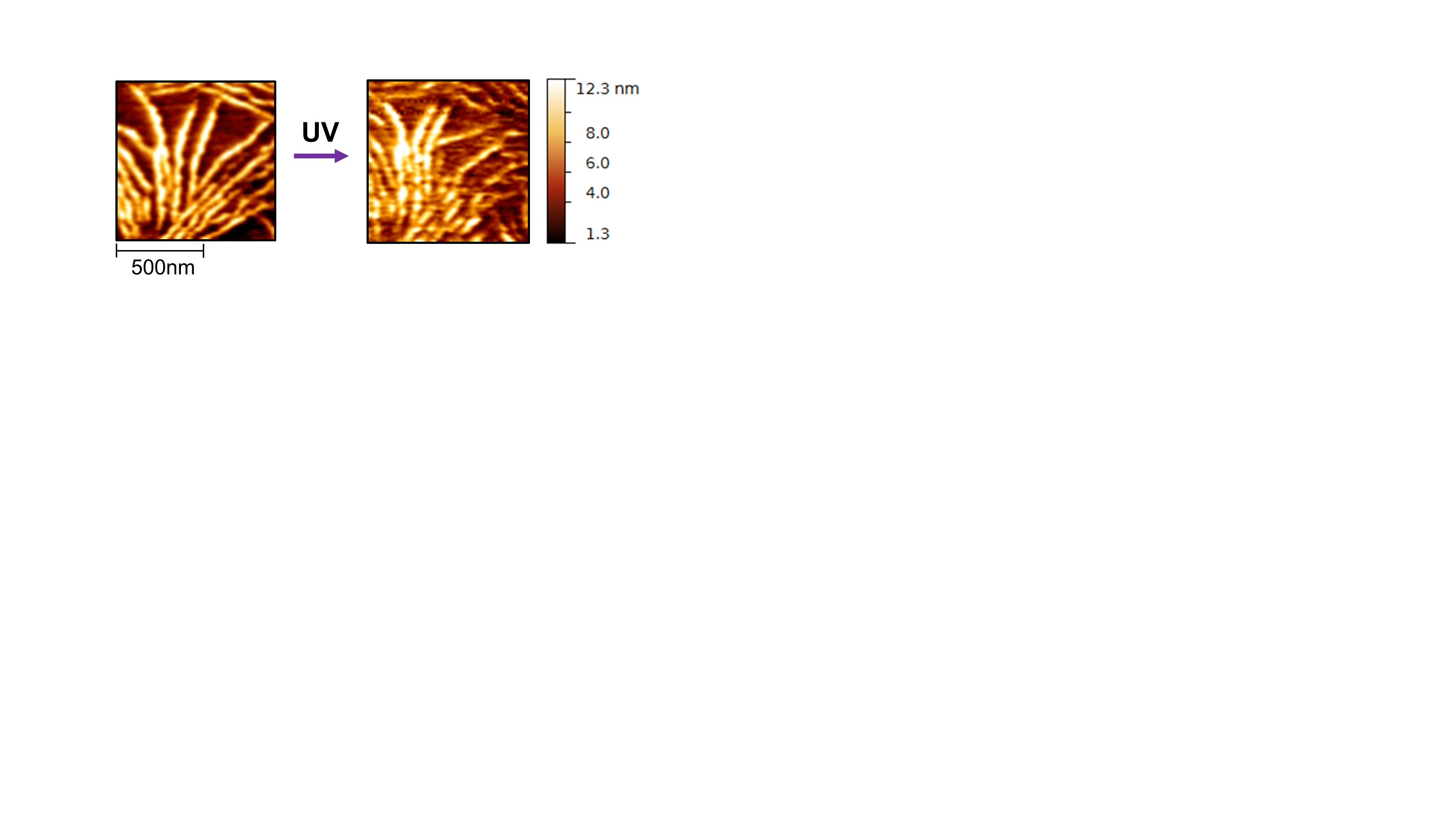


**Figure S3:** AFM images of the same sample area recorded in height mode before (left) and after (right) 5 min of UV-irradiation (30 mW/cm^2^).

The overall morphology remains unaffected. In some areas of the image small changes, *i.e.*, displacement of the nanofibers, can be seen. At least one of the straight nanofibers from the left image is clearly bent after UV-irradiation. Light-induced movement of single azobenzene polymers of **P1**-type has been observed before.^3^ However, we are not aware of reports about light-induced movements of supramolecular nanofibers at the mesoscale. For experimental reasons we were not able to monitor several switching cycles with AFM or to perform a detailed statistical analysis of the light-induced nanofiber motions.

**Spectral shift during photoisomerization**

The spectral position of the
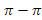
* transition band at different times during the *E-Z* photoisomerization process has been extracted from the time-resolved differential reflectance spectra shown in Figure 2b.

**
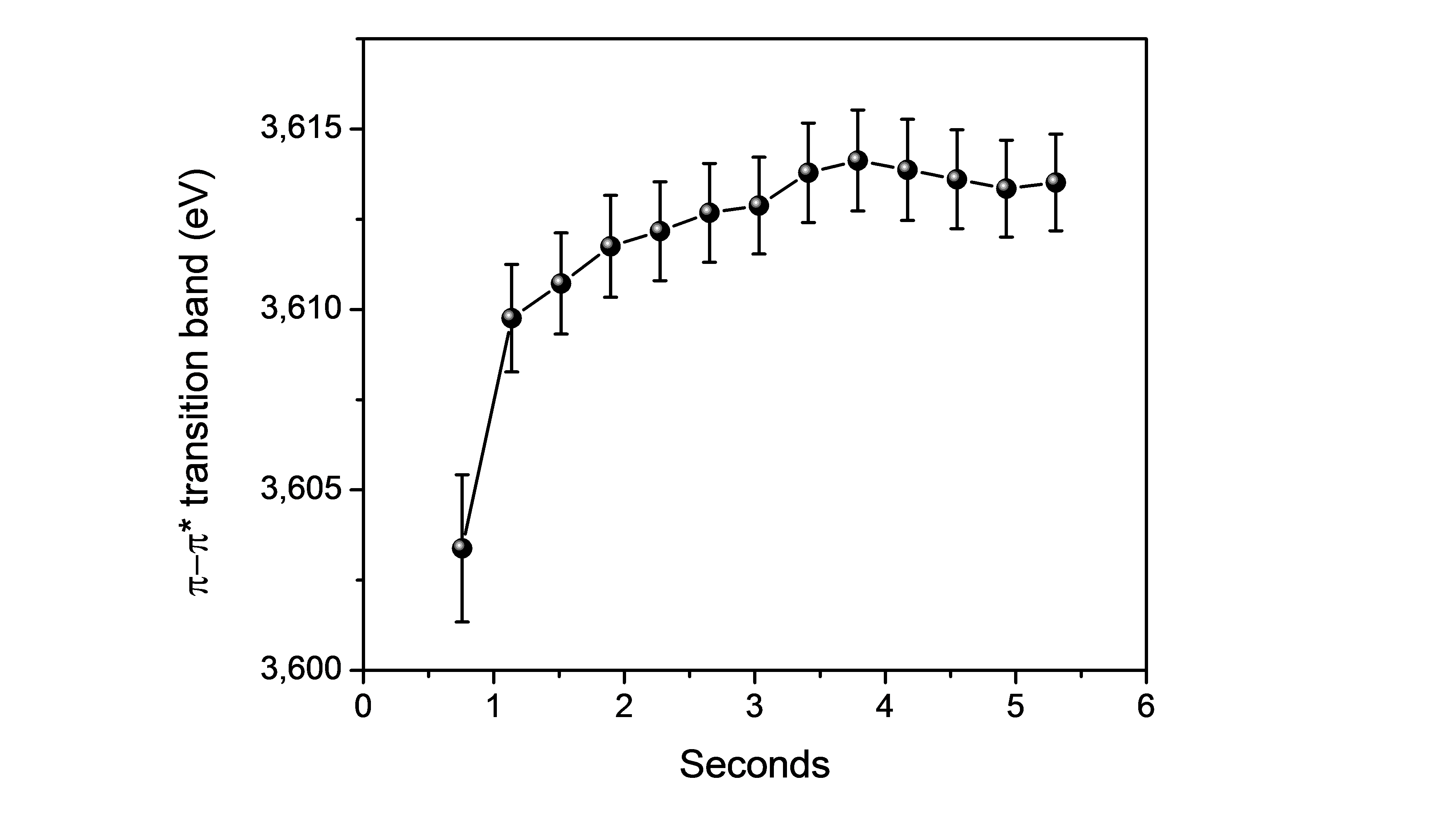
**

**Figure S4:** Spectral shift of the
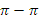
* transition band characteristic for *E* azobenzene, during *E-Z* photoisomerization.

**Simulated differential reflectance spectroscopy (DRS)**

We find deviations of a monoexponential kinetics in the real-time DRS measurement. In order to prove that this non-monoexponential behavior is intrinsic to the *Z* to *E* isomerization, it has to be shown that the *Z*-fraction is linearly dependent on the DRS signal Δ*R/R*. We therefore simulated the DRS signal of a **P1** film on silicon using a transfer matrix algorithm that is described elsewhere (see Figure S4a).^4^ This calculation indeed shows that around the π-π* absorption band, the DRS signal has a linear dependence on the fraction of *Z*-azobenzene as shown in Figure S4c.

**
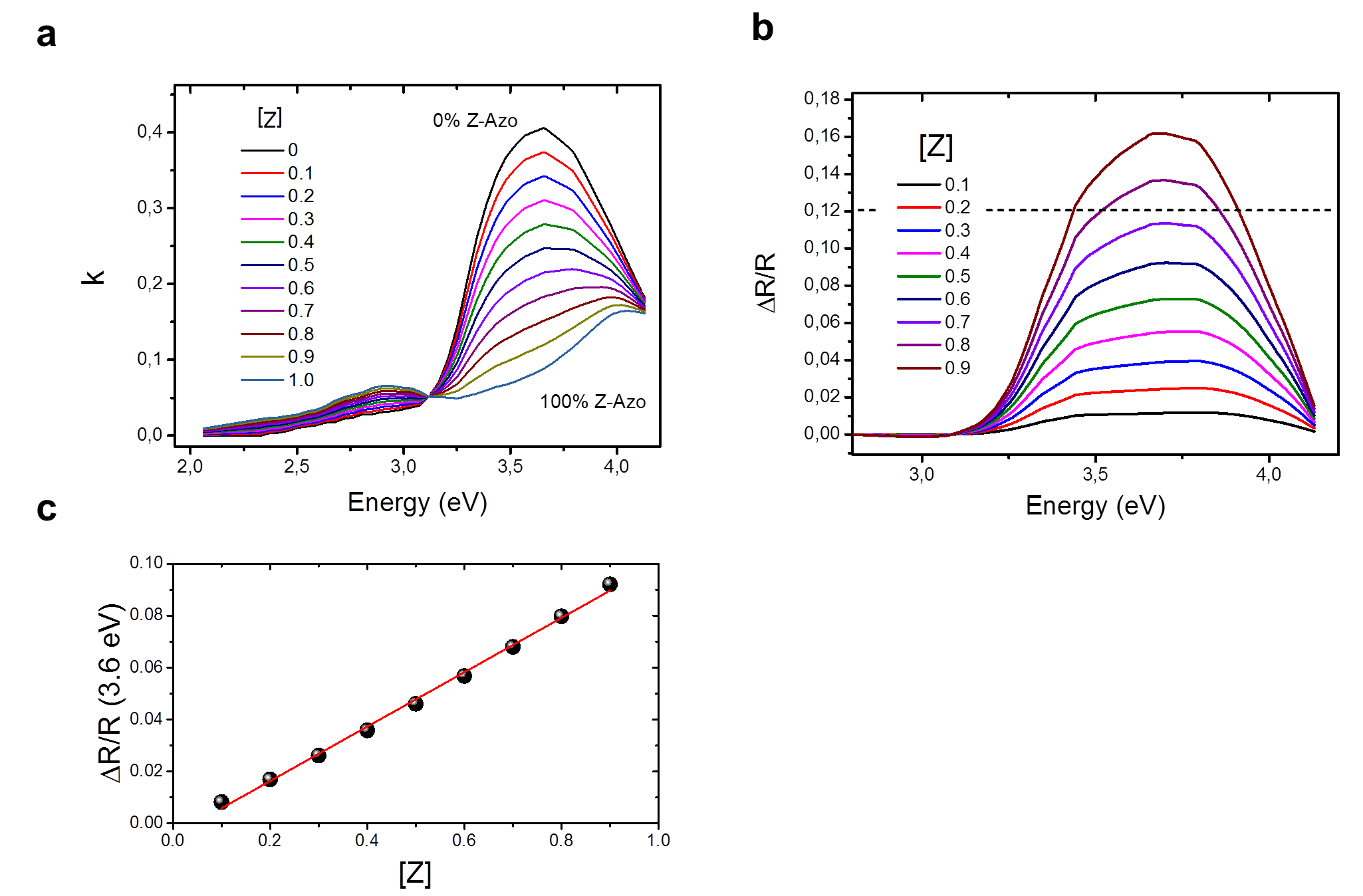
**

**Figure S5:** (a) Extinction coefficient *k* of the azobenzene oligomer film for different fractions of *Z*-azobenzene in the film. (b) Simulated differential reflectance spectra during *E* 🠢 *Z* conversion of **P1** on silicon covered with a thin layer of native silicon oxide. (c) Relation between the DRS signal and the fraction of *Z-*azobenzene in the film plotted for a photon energy of 3.6 eV.

The extinction coefficient *k* of **P1** both in the *E*- and in the *Z*-configuration was determined from absorption spectroscopy measurements in solution. For the simulation of the real-time DRS spectra during switching, the extinction coefficients of mixtures of *E*- and *Z*-**P1** were linearly interpolated between the values of pure *E*-**P1** and pure *Z*-**P1** (see Figure S4a). The molar extinction coefficient in solution was converted into the extinction coefficient in the film using the unit cell volume of **P1** polymers in thin films determined in ref. 2. The anisotropic alignment of the azobenzene chromophores in the fibrils was taken into account as the *E*-azobenzene transition dipole lies in the plane marked by the two phenyl rings.^5^ Consequently, the extinction coefficient for unpolarized light under normal incidence of the lying-down azobenzene chromophores in the fibrils is about twice as large as the extinction coefficient of azobenzene chromophores that are isotropically distributed in solution (upright standing azobenzene-oligomers can be excluded due to the limited height of the nanofibers).

The spectral shape of the refractive index *n* of *E*- and *Z*-**P1** was calculated from *k* using the Kramers-Kronig relation. Since the extinction coefficient of **P1** was only known in a finite energy interval between 2 eV and 6 eV, the refractive index *n* determined by the Kramers-Kronig relation might have an offset
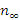
 due to additional resonances at energies above 6 eV. Also, within an effective medium approximation for the film consisting of a mixture of azobenzene chromophores, alkyl chains and air between the aggregates, we scaled the refractive index using a free parameter within physically reasonable boundaries to reproduce the experimental DRS data. Furthermore, a thin transparent adlayer (
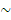
 5 nm) modelling the silicon oxide interface was included in the model for a fit of the DRS signal (The introduction or removal of this adlayer had no influence on the linear relation between the DRS signal and the fraction of *Z*-azobenzene, but improved the fit).

Figure S4b displays the resulting simulated DRS spectra during *E* to *Z* conversion of azobenzene. For increasing *Z*-fraction, the reflectivity increases in the region around 3.6 eV as observed in the experimental DRS data. At 3.6 eV, where the kinetics is evaluated, the DRS signal is to a good approximation linearly dependent on the fraction of *Z*-azobenzene (see Figure S4c). Using the simulated relation between Δ*R* and *Z,* we checked that the deviation from monoexponential kinetics cannot be quantitatively explained by the slight nonlinearity observed in Figure S4c.

**Analytical fits of the real-time DRS data**

The measured absorbance
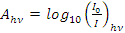
 of the thin film at a fixed wavelength
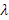
 can be written as a linear combination of the absorbance of *E-* and *Z*-isomers
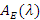
 and
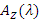
 at the given wavelength
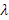
.


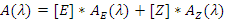
 (S3)

Here
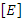
 and
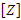
 denote the fraction of *E*-azobenzene and *Z*-azobenzene in the thin film, respectively. In the absence of a third photoactive azobenzene species the sum of the relative amount of *E*-azobenzene and *Z*-azobenzene will be constant throughout the isomerization reaction:


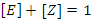
 (S4)

Combining equations (S3) and (S4) yields that the optical absorbance at the given wavelength
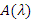
 has a linear dependence on the fraction of *Z*-azobenzene in the film and therefore also the reflectance
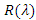
 behaves linearly.


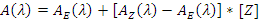
 (S5)

Consequently the differential reflectance
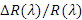
 is directly proportional to the change of *Z-*azobenzene fraction in the film.


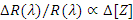
. (S6)

Note that
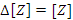
 if all azobenzenes are initially in the thermodynamically stable *E-*configuration.

Strictly speaking, eq. (S6) only holds if the absorption of the two isomers does not shift during the isomerization reaction. In general, this is not true in the solid state. The absorption spectrum of aggregated azobenzene molecules can be strongly shifted with respect to the absorption spectrum of isolated azobenzene molecules due to excitonic coupling between the azobenzene chromophores.^6^ Since the aggregation type may change depending on the isomeric state of azobenzenes one has to consider the effect that the absorbance of the two azobenzene isomers
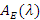
 and
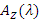
 may also depend on
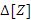
.^5^

However, scaling all the curves in Figure 2b to the same amplitude shows that the azobenzene absorption lines do not shift significantly during the isomerization reaction. One possible explanation is that π-stacking of azobenzene chromophores with azobenzene chromophores of adjacent oligomers is not possible due to the large dihedral angle between the azobenzene chromophores and the phenyl-rings on the one hand, and the long alkyl side chains on the other hand. Optical absorption spectroscopy performed on multilayer films of long **P1** polymers also showed no significant shift of the absorption line in the solid state with respect to its position in solution.^2^ This also suggests that there is no π-stacking of azobenzene chromophores in thin films of **P1**.

The above given relation (see eq. S6) allows one to fit the real time DRS data analytically using rate equations such as eq. (1) that describe the fraction of *E*- and *Z*-azobenzene in the film. Figure S5 shows fits to the DRS data based on the modified rate equation


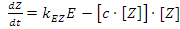
. (S7)

We simultaneously fitted ten datasets by numerically solving equation S7 under variation of the fit parameters
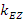
,
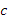
, and an overall scaling parameter. This scaling parameter [Z] / ΔR/R was set to 4.1 as this resulted in the best agreement of the model with the experimental data. This parameter serves as proportionality factor between the differential reflectance and the change of *Z-*fraction in eq. (S6). The additional parameters
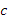
 and
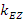
 were fitted, yielding
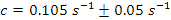
 and


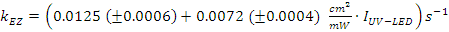
 (S8)

where
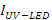
 denotes the intensity of the UV-LED in mW/cm^2^. For
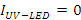
, the remaining *E* 🠢 *Z* photoisomerization rate
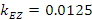
 has purely contributions from the probe light of the Xe-lamp.


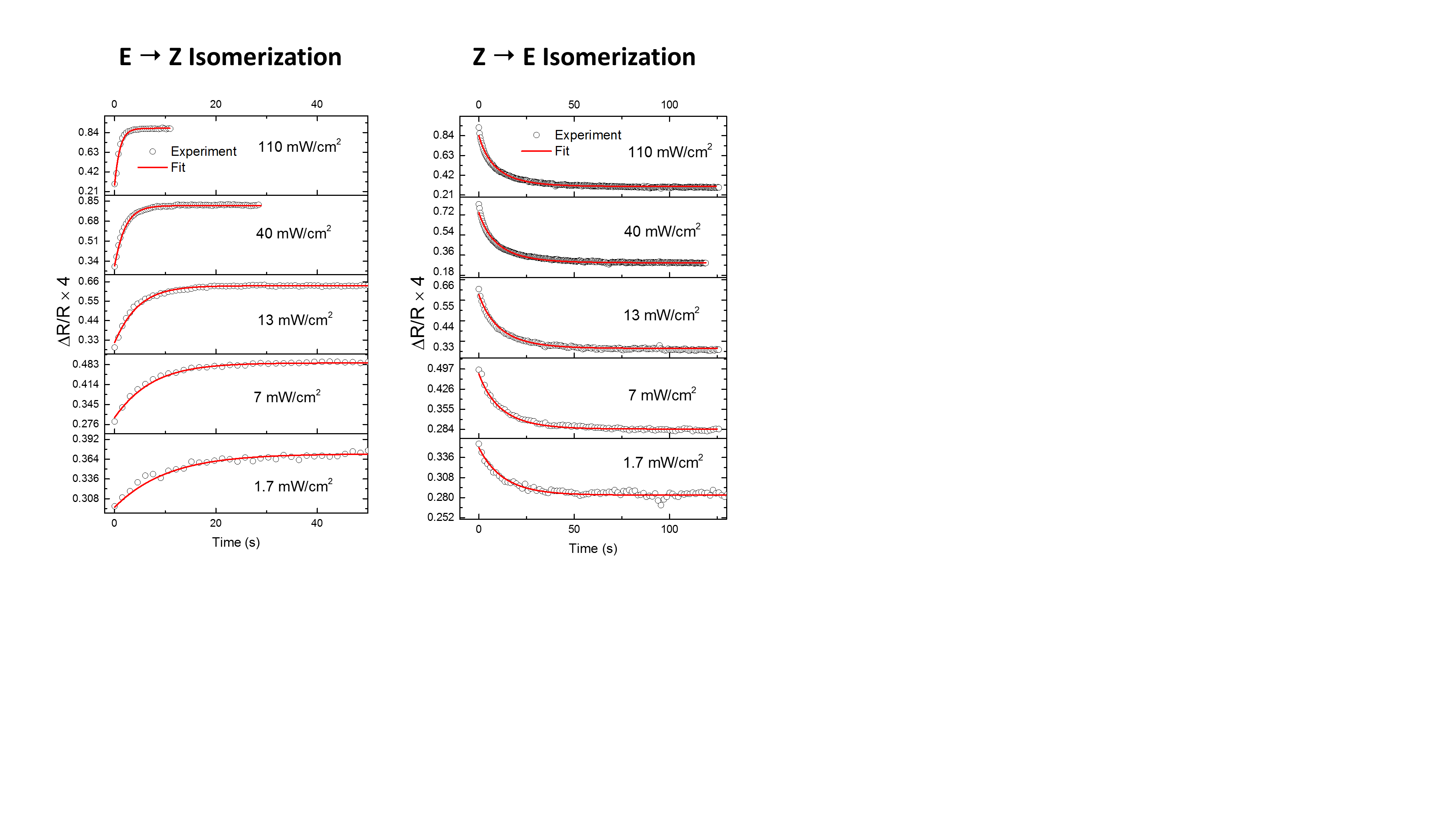


**Figure S6:** Real time DRS data during irradiation with UV-light (left) and visible light (right). Shown are five cycles of alternating irradiation with UV-light and visible light corresponding to five different intensities of the UV-LED. The red lines are analytical fits based on our modified rate equation model.

The photoisomerization rate
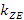
 = c
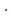
Z can be converted into the quantum yield of *Z* 🠢 *E* photoisomerization
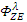
 via


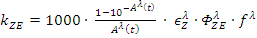
. (S9)

Here,
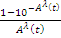
 is the photokinetic factor which in the case of an azobenzene layer with a thickness of about 3 nm and
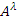
 < 0.01 can be assumed to be 2.3 to a good approximation^7^,
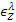
 denotes the molar extinction coefficient of *Z*-azobenzene and
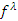
 is the photon flux. Since the *Z* 🠢 *E* photoisomerization is induced via n-π* excitation by blue light (400 nm – 500 nm) from our Xe-lamp, we only need to take this spectral region into account. The Xe-lamp has a total output of about 30 mW/cm^2^ in the region between 400 nm and 500 nm from which the photon flux can be directly calculated. For the molar extinction coefficient in this region, we took the respective value of pure *Z*-azobenzene in solution (
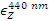
 = 1250 L
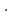
 mol^-1^
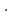
 cm^-1^ in n-hexane) as a first approximation. We observed no spectral shift of the azobenzene absorption in the nanofibers with respect to its absorption in solution which suggests that the absorption behaviour might be similar. Also, since the azobenzene units are electronically decoupled in the oligomers, a comparison with isolated azobenzene chromophores is reasonable. For the estimation, an averaged extinction coefficient between 400 nm and 500 nm of 600 L
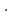
 mol^-1^
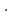
 cm^-1^ was used as input.

Using the modification that was introduced earlier,
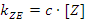
, we get a linear relation between
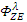
 and
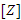
. Following the lowest UV-irradiation intensity leading to
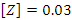
, we get
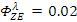
 and following the highest UV-irradiation intensity, leading to
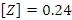
, we get
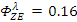
 as approximate values for the quantum yield.

**DRS at elevated temperatures**

We performed DRS at three different temperatures to investigate the temperature dependence of the cooperative switching behaviour. Figure S6a shows measurements of the differential reflectance during several switching cycles using different UV-LED intensities at 25°C, 120°C and 180°C. All measurements were done for the same **P1** oligomer thin film and performed on the same sample spot. After heating up the sample with a heating stage we waited for 1h for the sample to equilibrate its temperature. Note that at higher temperatures more azobenzenes are switched from *E* to *Z* under the same irradiation conditions as indicated by the higher ΔR/R level of the photostationary state (see Figure S6b). This also shows that the Arrhenius type behaviour of the thermal back switching is not the dominant thermal effect here.

**
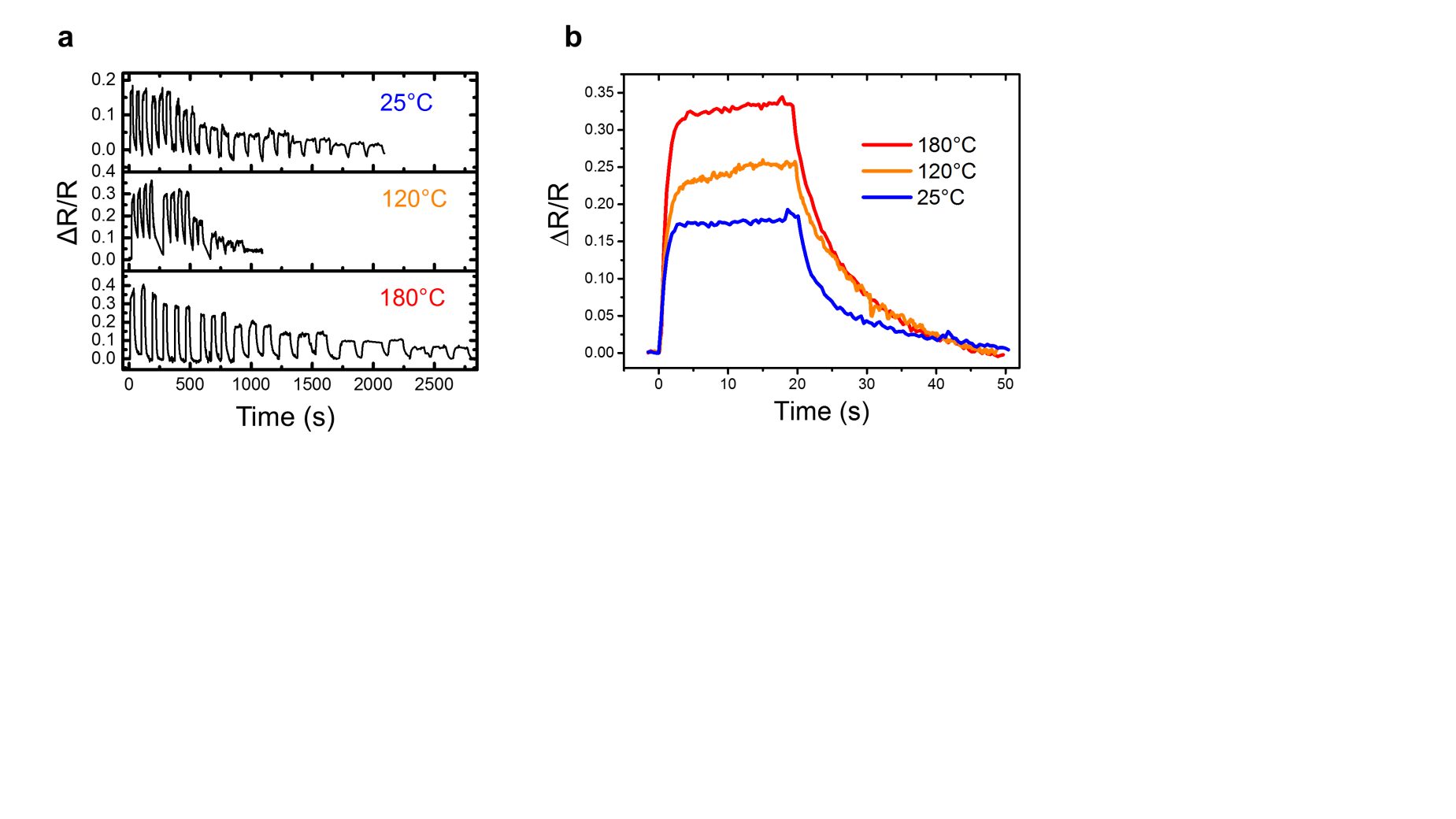
**

**Figure S7:** (a) Real-time DRS data of **P1** oligomers during several switching cycles at three different temperatures. (b) Direct comparison of real-time DRS at 25°C, 120°C and 180°C during a single switching cycle.

**References**

1. Bléger, D. *et al.* Light-orchestrated macromolecular ‘accordions’: reversible photoinduced shrinking of rigid-rod polymers. *Angew. Chem. Int. Ed.* **50,** 12559 (2011).

2. Weber, C. *et al.* Light-Controlled ‘Molecular Zippers’ Based on Azobenzene Main Chain Polymers. *Macromolecules* **48,** 1531 (2015).

3. Lee, C., Liebig, T., Hecht, S., Bléger, D. & Rabe, J. P. Light-Induced Contraction and Extension of Single Macromolecules on a Modified Graphite Surface. *ACS Nano* **8,** 11987 (2014).

4. Burkhard, G. F., Hoke, E. T. & McGehee, M. D. Accounting for interference, scattering, and electrode absorption to make accurate internal quantum efficiency measurements in organic and other thin solar cells. *Adv. Mater.* **22,** 3293 (2010).

5. Moldt, T. *et al.* Tailoring the Properties of Surface-Immobilized Azobenzenes by Monolayer Dilution and Surface Curvature. *Langmuir* **31,** 1048 (2015).

6. Gahl, C. *et al.* Structure and excitonic coupling in self-assembled monolayers of azobenzene-functionalized alkanethiols. *J. Am. Chem. Soc.* **132,** 1831 (2010).

7. Sekkat, Z. & Knoll, W. *Photoreactive Organic thin Films in the Light of Bound Electromagnetic Waves*. (Academic Press, 2002). doi:10.1002/9780470133538.ch2
